# Tumor Cell-Based Vaccine Generated With High Hydrostatic Pressure Synergizes With Radiotherapy by Generating a Favorable Anti-tumor Immune Microenvironment

**DOI:** 10.3389/fonc.2019.00805

**Published:** 2019-08-28

**Authors:** Christoph Seitz, Michael Rückert, Lisa Deloch, Eva-Maria Weiss, Sebastian Utz, Marika Izydor, Nina Ebel, Eberhard Schlücker, Rainer Fietkau, Udo S. Gaipl, Benjamin Frey

**Affiliations:** ^1^Department of Radiation Oncology, Friedrich-Alexander-Universität Erlangen-Nürnberg, Universitätsklinikum Erlangen, Erlangen, Germany; ^2^Department of Psychiatry and Psychotherapy, Friedrich-Alexander-Universität Erlangen-Nürnberg, Universitätsklinikum Erlangen, Erlangen, Germany; ^3^Institute of Process Machinery and Systems Engineering, Friedrich-Alexander-Universität Erlangen-Nürnberg, Erlangen, Germany; ^4^Department of Cardiac Surgery, Friedrich-Alexander-Universität Erlangen-Nürnberg, Universitätsklinikum Erlangen, Erlangen, Germany

**Keywords:** radiotherapy, immunotherapy, tumor cell-based vaccine, high hydrostatic pressure, malignant melanoma, colorectal carcinoma, tumor-infiltrating leukocytes, tumor microenvironment

## Abstract

Dendritic cell (DC)-based vaccines pulsed with high hydrostatic pressure (HHP)-inactivated tumor cells have been demonstrated to be a promising immunotherapy for solid tumors. We focused on sole injection of tumor cells that were inactivated by HHP and their combination with local radiotherapy (RTx) for *in vivo* induction of anti-tumor immune responses. HHP-treatment of tumor cells resulted in pre-dominantly necrotic cells with degraded DNA. We confirmed that treatments at 200 MPa or higher completely inhibited the formation of tumor cell colonies *in vitro*. No tumor growth was seen *in vivo* after injection of HHP-treated tumor cells. Single vaccination with HHP-killed tumor cells combined with local RTx significantly retarded tumor growth and improved the survival as shown in B16-F10 and CT26 tumor models. In B16-F10 tumors that were irradiated with 2 × 5Gy and vaccinated once with HHP-killed tumor cells, the amount of natural killer (NK) cells, monocytes/macrophages, CD4+ T cells and NKT cells was significantly increased, while the amount of B cells was significantly decreased. In both models, a trend of increased CD8+ T cell infiltration was observed. Generally, in irradiated tumors high amounts of CD4+ and CD8+ T cells expressing PD-1 were found. We conclude that HHP generates inactivated tumor cells that can be used as a tumor vaccine. Moreover, we show for the first time that tumor cell-based vaccine acts synergistically with RTx to significantly retard tumor growth by generating a favorable anti-tumor immune microenvironment.

## Introduction

In recent years, cancer immunotherapy has revived. It comes in a variety of forms, including checkpoint inhibitors, targeted antibodies, adoptive cell transfer, tumor-infecting viruses, cytokines, adjuvants, and cancer vaccines. Cancer vaccines aim specifically to activate the immune system in cancer patients (1). As dendritic cells (DCs) link the innate and adaptive immune system as powerful antigen-presenting cells, they were used as cancer vaccines in several clinical trials. DC-based immunotherapy has been demonstrated to be safe and capable of inducing anti-tumor immunity. Long-term survival in advanced melanoma patients undergoing DC vaccination is similar to ipilimumab-treated patients (2). Nevertheless, the response rates are often low. Improved vaccines with higher immunogenicity and particularly combination with other tumor therapies should therefore be implemented (3).

High hydrostatic pressure (HHP)-treatment is an innovative method for the generation of whole cell-based tumor vaccines. Although HHP has been mainly used in the food industry for processing and preserving meat and other food to avoid thermal treatment (4). Though HHP is known to denature proteins, it doesn't affect covalent bonds, meaning that the proteins' primary and secondary structure is maintained, whereas their tertiary and quaternary structure is changed (5). Urbanova et al. showed that HHP-treatment of tumor cells affects the antigenic pool and that loading of DCs with HHP-killed tumor cells can induce CD8+ T cell responses *in vitro* (6). Fucikova et al. demonstrated that HHP-treatment induces immunogenic cancer cell death in human tumor cells and that interaction of HHP-killed cancer cells with DCs results in phagocytosis of the tumor cells and activation of the DCs (7). DCs pulsed with HHP-killed cancer calls can be used as cancer vaccine (8). Based on these data, *ex vivo* HHP-killed tumor cell-loaded DCs are currently being tested in clinical trials as therapeutic cancer vaccines. For this, patient's monocyte-derived DCs pulsed with HHP-killed allogeneic tumor cell lines (DCVAC) are used to treat prostate, ovarian and lung cancer (NCT03514836, NCT03905902, NCT02470468). One has to stress that such tumor vaccination is well-combinable with chemotherapy (9).

We have aimed to test whether sole injection of HHP-killed tumor cells without DCs can also be used as a cancer vaccine in a multimodal approach together with RTx, hypothesizing that under distinct *in vivo* micro-environmental conditions such inactivated tumor cells are taken up by endogenous DCs. We already demonstrated in previous work that murine CT26 tumor cells are effectively inactivated by HHP-treatment and that specific IgG antibodies against tumor cells were significantly increased after immunization of mice with HHP-treated tumor cells (10). This work gave first hints that sole injection of HHP-killed tumor cells is capable of triggering anti-tumor immune responses *in vivo*. In variance to the approach of DC pulsed vaccines, we use syngeneic rather than allogeneic tumor cells for vaccination. This syngeneic vaccine mimicking in cancer patients autologous vaccine from their own tumor cells should contain all potentially relevant tumor-associated antigens (TAAs) for a particular patient (11).

It should be stressed that HHP treatment fulfills the main requirements for clinical vaccine: it effectively inactivates tumor cells, it has no intrinsic toxicity, it does not destroy the immunogenicity of the tumor cells and it can be applied with legal and GMP-compliant requirements. Further, it is further a highly reproducible and easy to apply method (12). Therefore, HHP is advantageous to other preparation methods such as heat killing, radiation, or freeze-thaw approaches.

We performed our pre-clinical studies with two broadly used B16-F10 melanoma and CT26 colorectal cancer models. Although malignant melanoma is an aggressive disease with rising incidence and high resistance to classical therapy, targeted therapies and immune therapy have significantly improved the treatment of patients with advanced malignant melanoma in recent years (13). In colorectal cancer, the proportion of patients with an immunosuppressive tumor microenvironment is high, again calling for combination therapies that modulate the immune system (14).

Emerging evidence suggests that radiotherapy (RTx) is capable of activating the patient's immune system by acting as an *in situ* cancer vaccine (15, 16). RTx modifies the phenotype of the tumor cells and the tumor microenvironment (17). It however results in both, immune activation and immune suppression (18). Therefore, the combination of RTx with immunotherapy has the potential to induce regression of tumors, even outside of the radiation field (19).

It has become evident that in established cancers anti-tumor vaccines will require co-treatments to overcome immune evasion (20). RTx might act as adjuvants for the vaccine and this combination might be effective in generating anti-tumor immune responses. Here we show for the first time that a single vaccination with HHP-killed tumor cells combined with local RTx significantly retards tumor growth and improves survival of tumor-bearing mice by generating a favorable anti-tumor immune environment as analyzed in B16-F10 and CT26 tumor models.

## Materials and Methods

### Cell Lines and Cell Culture

B16-F10 melanoma and CT26 colon carcinoma cells were both obtained from ATCC (Manassas, VA, USA). The tumor cells were grown up to a maximum confluence of 80% at 37°C, 5% CO_2_, and 95% humidity, in RPMI 1640 (Sigma Aldrich, Munich, Germany) with the addition of 10 % fetal bovine serum (FBS, Biochrom AG, Berlin, Germany) and 1% penicillin-streptomycin (PenStrep, Gibco, Carlsbad, USA).

### High Hydrostatic Pressure Treatment

After detaching of the adherent tumor cells, the cell suspension was transferred into cryovials (Greiner Bio-one, Frickenhausen, Germany). The vials were filled completely (2.5 ml) and closed tightly by avoiding any air bubbles. Afterwards the vials were sealed with Parafilm™ (American National Can, Chicago, USA) to prevent leaking.

The equipment for HHP-treatment (Supplemental Figure 1) was provided by the “Institut für Prozessmaschinen und Anlagentechnik” (iPAT, Friedrich-Alexander-Universität Erlangen-Nürnberg). For pressurizing the tumor cells, the cryovial with the cell suspension was put into the autoclave (1). Pressure that was built up at a velocity of around 5 MPa/s by a manual spindle press (2) in addition to a pneumatic pump (3) was transmitted to the autoclave via a system of metal tubes (4) containing pressure transmitting fluid (hydraulic oil Ultra-Safe 620, Petrofer, Hildesheim, Germany). According to Pascal's law, pressure which is generated and transmitted by the transmitting fluid acts to the same amount on the cells filled in the cryovials. The fluid is stored in a reservoir (5) that is attached to the aperture and the pressure can be recorded via a digital manometer (6). The pressure is maintained and released by several switches (7) in the aperture. Since different pressure levels showed promising results for inactivation of tumor cells in earlier studies (7, 10, 21–23), we also first tested pressure from 100 to 500 MPa at a compression time of 300 s for some *in vitro* examinations of the vaccine. According to the 3Rs concept for more ethical use of animals in testing, namely replacement, reduction and refinement, we focused on whole tumor cell-based vaccines generated with 200 MPa for the *in vivo* tumor models. Generally, after pressurizing, the tumor cells were first re-cultivated in cell culture flasks (Greiner Bio-one, Frickenhausen, Germany).

### Cell Death Detection by AnnexinA5/Propidium Iodide Staining

For analyses of cell death forms by flow cytometry (EPICS XL MCL, Beckman Coulter, Brea, USA), HHP-treated tumor cells were suspended in 400 μl Ringer (B. Braun, Melsungen, Germany) and stained with FITC-labeled AnnexinA5 (AnxA5; 0.2 μl, Geneart, life technologies, Regensburg, Germany) and propidium iodide (PI; 0.4 μl, Sigma Aldrich, Munich, Germany) according to the protocol of Vermes et al. (24). AnxA5-negative/PI-negative cells were considered as viable ones, AnxA5-positive/PI-negative as apoptotic cells and AnxA5-positive/PI-positive cells as necrotic ones.

### Cell Cycle and SubG1 DNA Content Analyses With Propidium Iodide

1 × 10^6^ tumors cells were fixed in 70 % ethanol and incubated at −20°C for at least 20 min. Afterwards, a solution containing Triton X-100 (Sigma Aldrich, Munich, Germany), 200 μg/ml RNase (Biochemica, Buchs, Germany), and 5 μg/ml PI was added at room temperature for at least 30 min. The cell cycle phases were consecutively analyzed by flow cytometry. Apoptotic and secondary necrotic cells that lost their nuclear DNA content due to DNA fragmentation show subG1 DNA content (25).

### Monitoring of the Clonogenicity of HHP-Treated Cells *in vitro* and *in vivo*

*In vitro*, the pressurized tumor cells were plated in multiplicates at increasing concentrations in petri dishes (BD Falcon, New York, USA) and cultivated for 10 days. After staining the cells with 3 ml methylene blue (Sigma Aldrich, Munich, Germany), colonies consisting of more than 50 cells were scored (26). For *in vivo* analysis, a suspension of 2 × 10^6^ treated tumor cells in Ringer's solution was injected subcutaneously into mice. The subsequent tumor growth was analyzed up to 39 days after injection of the tumor cells.

### Multimodal Treatment of Tumor-Bearing Mice

All animal experiments were conducted according to the guidelines of the “Federation of European Laboratory Animal Science Associations” (FELASA) and the “Gesellschaft für Versuchstierkunde” (GV-SOLAS) and were authorized by the government of Mittelfranken/Unterfranken. C57BL/6 mice were inoculated subcutaneously with 1 × 10^6^ viable B16-F10 melanoma cells. After 8 days, when a visible and vascularized tumor was established, the mice were either locally irradiated with 2 × 5Gy at day 8 and 10, subcutaneously vaccinated next to the tumor with 5 × 10^6^ 24 h-aged HHP-treated cells without any additional adjuvant on day 11, locally irradiated plus vaccinated, or left untreated. For the induction of CT26 tumors, Balb/c mice were injected subcutaneously with 1.2 × 10^6^ viable CT26 colon carcinoma cells. In this tumor model, palpable tumors were established after 14 days. Beginning on that day, the treatment was conducted in the same scheme as for the B16-F10 cells. Since pressure of 200 MPa showed promising results *in vitro* and in former studies (10, 12, 23) and according to the 3Rs concept for more ethical use of animals in testing, this pressure level was used for the *in vivo* experiments. Tumor growth was determined with an electronic caliper. The tumor volume was calculated by the formula VTumor = ½ · (L · B2) (27). Mice were sacrificed whenever the tumor volume exceeded 1,600 mm^3^ or the well-being of the mouse was reduced according to approved criteria. A PRIMART linear accelerator (Siemens, Munich, Germany) was used for RTx. The local irradiation of the tumor-bearing mice was performed closely resembling the clinical situation as previously established and applied by our group (28, 29).

### Immune Phenotyping of Tumors and Blood

Tumor samples and whole blood for immune phenotyping by multicolor flow cytometry were taken in a group of mice on day 7 after first irradiation. Erythrocyte lysis of blood samples was performed with a TQ-Prep™ Workstation (Beckman Coulter, Brea, USA) prior to the antibody staining. Single cell suspensions from tumors were obtained with the Tumor Dissociation Kit and the gentleMACS™ Dissociator according to the manufacturer's instructions (Miltenyi Biotec, Bergisch Gladbach, Germany). To enrich tumor samples for tumor-infiltrating leukocytes (TIL), CD45 MicroBeads (Miltenyi Biotec, Bergisch Gladbach, Germany) for subsequent separation with MACS^®^ Technology according to the manufacturer's instructions (Miltenyi Biotec, Bergisch Gladbach, Germany) were used. The following antibodies were applied for the staining of both, tumor and blood samples: CD3e V450, CD4 FITC, Ly-6C FITC, CD11c BV510, CD19 APC-Cy7, and Ly6G PE-Cy7 (all from BD Biosciences, Franklin Lakes, USA), CD49b APC, PD-1 PE/Dazzle 594, CD8a BV605, Zombie NIR and Zombie Aqua (all from Biolegend, San Diego, USA), CD11b APC, CD45.2 PerCP-Cy5.5, MHC-II (I-A/I-E) eFluor 450 and γδTCR PE (all from eBioscience, San Diego, USA). All samples were acquired with a CytoFLEX S flow cytometer (Beckman Coulter, Brea, USA) and analyzed with the Kaluza software (Beckman Coulter, Brea, USA). To calculate the concentration of tumor infiltrating leukocytes (cells/g tumor), tumors were weighed prior to dissociation.

### Statistical Analysis

The types of statistical test for data analyses are depicted in the figure legends. Results were considered statistically significant for *p* < 0.05 (^*^) and highly significant for *p* < 0.01(^**^).

## Results

### High Hydrostatic Pressure Treatments at 200 MPa or Higher Pre-dominantly Induce Necrosis in Tumor Cells

**Figure 1:**
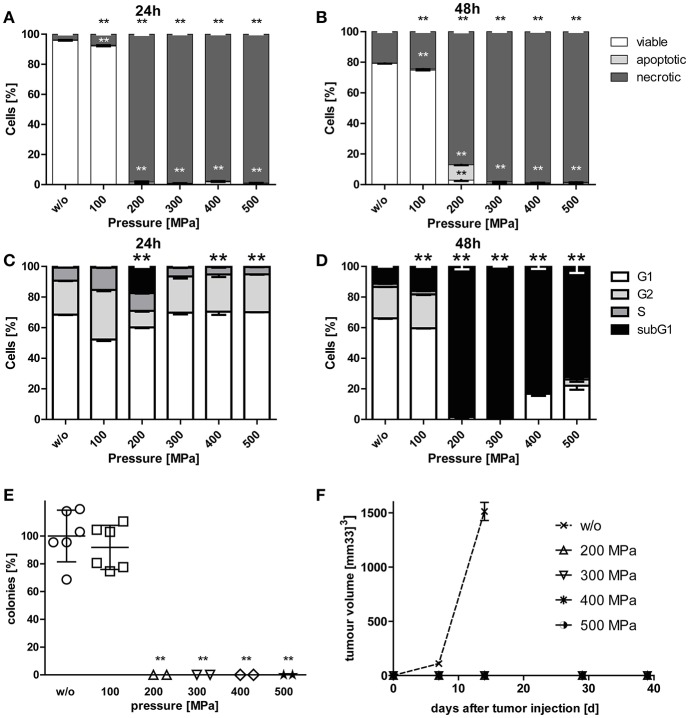
Cell death and clonogenicity of B16-F10 melanoma cells following treatment with HHP. B16-F10 melanoma cells were treated with pressure at 100 MPa up to 500 MPa for 5 min and afterwards cultured for 24 **(A,C)** and 48 h **(B,D)**, respectively. The cells were either stained with AnxA5-FITC/PI **(A,B)** or PI in the presence of detergent **(C,D)** and cell death as well as cell cycle phases were analyzed by flow cytometry. The percentages of viable (AnxA5^−^/PI^−^), apoptotic (AnxA5^+^/PI^−^), and necrotic (AnxA5^+^/PI^+^) cells are displayed in **(A)** and **(B)**. The percentages of cells in the G1-, G2-, and S-phase as well as the subG1 DNA content are displayed in **(C)** and **(D)**. Data of three independent experiments are presented as mean ± SD. **(E)** shows the *in vitro* colony formation of HHP-treated B16-F10 melanoma cells. Single values, means, and SDs are presented. **(F)** displays the growth of syngeneic B16-F10 tumors in C57BL/6 mice after subcutaneous injection of 2 × 10^6^ HHP-treated tumor cells. Three mice were used for each treatment condition. Data are presented as mean ± SEM. w/o: mock-treated control. Significant values are determined by an unpaired, one-tailed Student's *t*-test with Welch's correction for unequal variances; ^**^p < 0.01 related to w/o.

While viability of B16-F10 melanoma cells was hardly affected by pressurizing with 100 MPa, treatments at 200 MPa or higher resulted in mostly necrotic tumor cells. Small percentages of apoptotic melanoma cells were observed when the tumor cells were treated with 200 MPa (Figures 1A,B). The latter pressure already resulted in degraded tumor DNA as early as 24 h after treatment. Almost all melanoma cells had degraded DNA 2 days after pressurizing if they were treated with pressure above 100 MPa (Figures 1C,D).

### High Hydrostatic Pressure Treatments at 200 MPa or Higher Effectively Inactivate Tumor Cells

To prove the inactivation of B16-F10 melanoma cells after HHP-treatment, their potential to form colonies *in vitro* (Figure 1E) and their potential for progression *in vivo* after having been injected into C57BL/6 mice was analyzed (Figure 1F). Treatments at 200 MPa or higher completely inhibited the formation of colonies *in vitro*. Further, no tumor growth was seen *in vivo* after tumor cell injection. Notably, pressure of 100 MPa is not sufficient to suppress colony formation of melanoma cells. Similar results were already previously observed for CT26 cells (23).

### Combination of RTx With Whole Tumor Cell-Based Vaccine Generated by HHP Significantly Retards Tumor Growth in C57BL/6 Mice and Increases Their Survival

**Figure 2:**
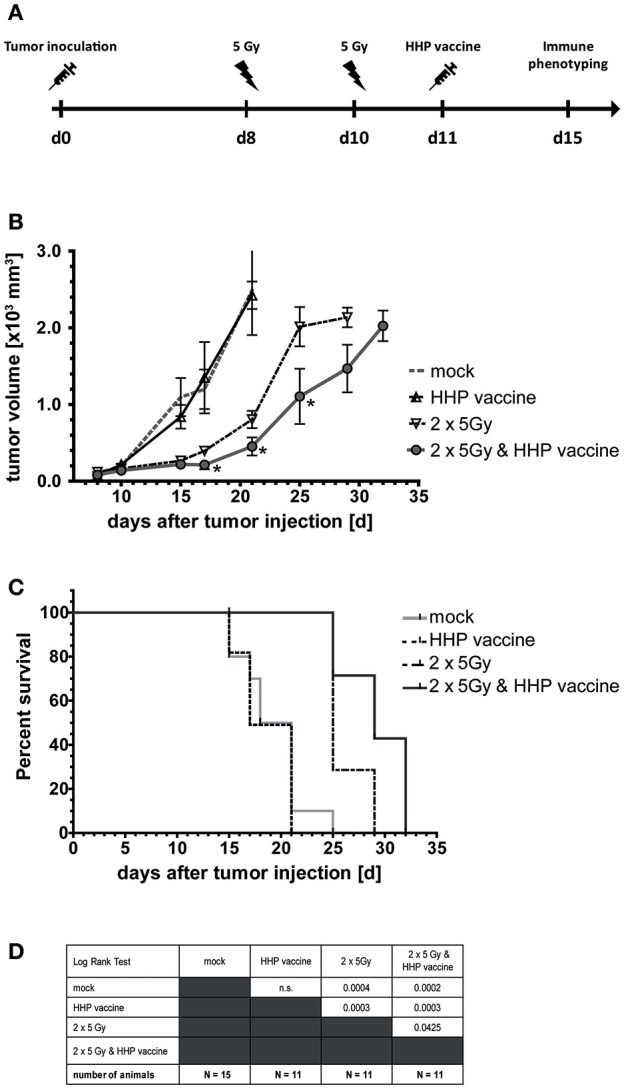
Impact of radiotherapy and whole tumor cell-based vaccine generated by HHP on B16-F10 melanoma growth and survival. **(A)** 1 × 10^6^ viable B16-F10 melanoma cells were injected subcutaneously into syngeneic C57BL/6 mice. The tumor-bearing mice were either left untreated (mock), vaccinated at day 11 with 5 × 10^6^ B16-F10 tumor cells that had been inactivated with 200 MPa (HHP vaccine), locally irradiated at day 8 and 10 after tumor implantation with a single dose of 5Gy per fraction (2 × 5Gy), or treated with both RTx (2 × 5Gy) and HHP vaccine. The tumor growth is displayed in **(B)** and the survival time of C57BL/6 mice after B16-F10 tumor implantation in **(C)**. When the tumor volume exceeded 1,600 mm^3^, the mice were euthanized. The number of mice in the treatment groups, as well as the results of the Log-Rank (Mantel-Cox) test concerning the survival **(C)** is displayed in **(D)**. Significant values are determined by an unpaired, one-tailed Student's *t*-test with Welch's correction for unequal variances. The normality of the values was confirmed by a Kolmogorov-Smirnov and Shapiro-Wilk test for each time point. Four animals of each group were sacrificed on day 15 to analyze the infiltrating immune cells; ^*^p < 0.05.

Eight and 10 days after tumor inoculation, the tumors were locally irradiated with 2 × 5Gy and vaccination with HHP-treated tumor cells was performed at day 11 (Figure 2A). Vaccination with HHP-treated cells was not sufficient to significantly slow-down the tumor growth (Figure 2B). At day 21 after tumor inoculation, all mice of the vaccination and control group had to be euthanized, because the tumor volume had exceeded 1,600 mm^3^. RTx resulted in significantly retarded tumor growth when compared to vaccinated or mock-treated animals. Vaccination with HHP-treated cells in addition to RTx at day 11 resulted in further significant tumor growth retardation and even at day 32 after tumor inoculation three animals could still be monitored. Similarly to the tumor growth reduction, the survival of the mice could be significantly improved by RTx alone, and was further significantly improved when RTx was combined with vaccination (Figures 2C,D).

### Combination of RTx With Whole Tumor Cell-Based Vaccine Generated by HHP Generates a Beneficial Immune Cell Infiltrate for Melanoma

**Figure 3:**
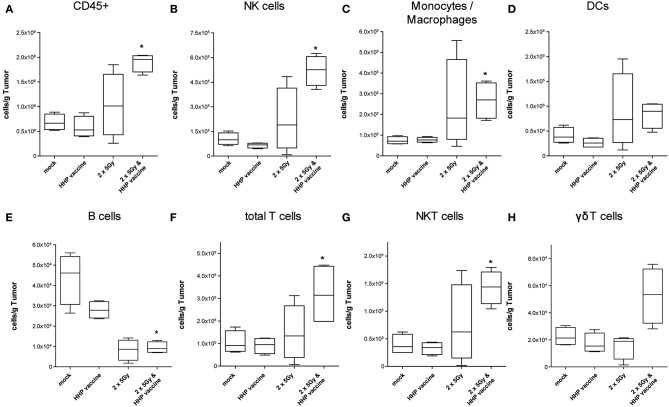
Impact of radiotherapy and whole tumor cell-based vaccine generated by HHP on infiltration of immune cells into B16-F10 tumors. Single cell suspensions of tumors from mice that had been treated with RTx (2 × 5Gy) and/or HHP vaccine (B16-F10 tumor cells that had been inactivated with 200 MPa) were prepared on day 15 after tumor inoculation. Multicolor flow cytometry was performed to detect tumor-infiltrating immune cell subtypes. The latter were identified as follows: all immune cells CD45+ **(A)**; natural killer (NK) cells CD49b+, CD3– **(B)**; Monocytes/Macrophages CD11b+, Ly-6C+ **(C)**; dendritic cells (DCs) MHC-II+, CD11c+ **(D)**; B cells CD19+ **(E)**; total T cells CD3+ **(F)**; NKT cells CD3+, CD49b+ **(G)**; γδT cells CD3+, γδTCR+ **(H)**. Data are presented as box plots showing the median and minimum to maximum values. *n* = 4; Mann-Whitney *U* test was used for statistical analyses compared to the control group; ^*^p < 0.05.

A sole vaccination of the mice with HHP-killed tumor cells did not affect infiltration of cells of the innate and adaptive immune system into B16-F10 tumors. RTx with 2 × 5Gy slightly, but not significantly enhanced the infiltration of NK cells, monocytes/macrophages, DCs and NKT cells. Only the combination of RTx with HHP vaccine significantly increased the total number of immune cells (CD45+) per gram of tumor, which were almost 3 fold higher compared to mock-treated controls (Figure 3A). The immune infiltrates primarily consisted of NK cells (CD3–, CD49b+; Figure 3B), monocytes or macrophages (CD11b+, Ly-6C+; Figure 3C) and T cells (CD3+; Figure 3F); about half of the latter being NKT cells (CD3+, CD49b+; Figure 3G). Although not as prominent and in lower absolute numbers, also DCs (MHC-II+, CD11c+; Figure 3D) and in particular γδT cells (CD3+, γδTCR+; Figure 3H) tended to be present in higher numbers after the combined treatment. In contrast to the other immune cell types, B cell (CD19+; Figure 3E) numbers were significantly reduced after RTx plus vaccination. No major alterations were found for neutrophil, eosinophil, basophil, and pDC infiltration (data not shown).

**Figure 4:**
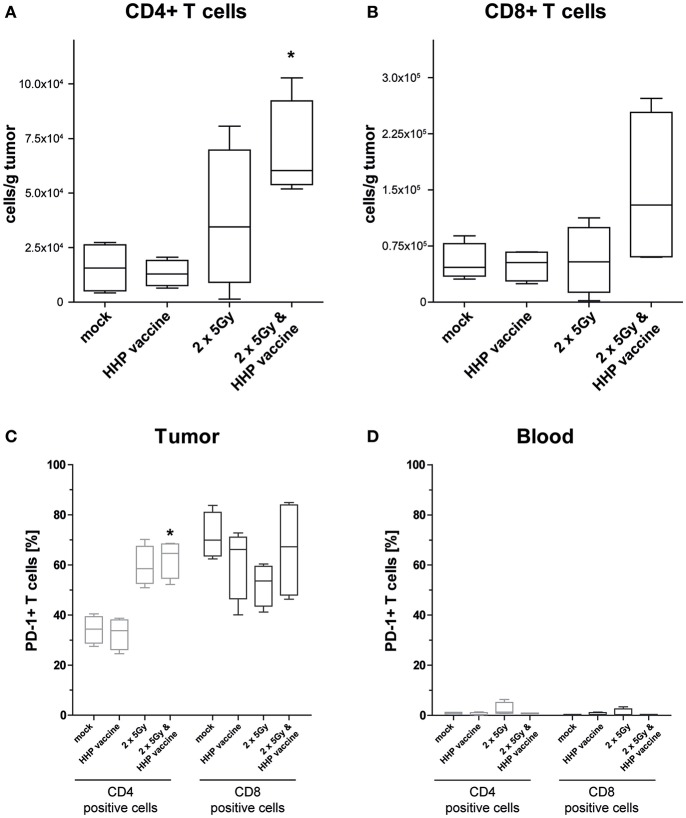
Impact of radiotherapy and whole tumor cell-based vaccine generated by HHP on infiltration of T cells into B16-F10 tumors and on PD-1 expression of T cells. Single cell suspensions of tumors from mice that had been treated with RTx (2 × 5Gy) and/or HHP vaccine (B16-F10 tumor cells that had been inactivated with 200 MPa) were prepared on day 15 after tumor inoculation. Multicolor flow cytometry was performed to detect tumor infiltrating CD4+ **(A)** and CD8+ T cells **(B)**, respectively. Further, expression of PD-1 on T cells being present in the tumors **(C)** and those circulating in blood **(D)** is shown. Data are presented as box plots showing the median and minimum to maximum values. *n* = 4; Mann-Whitney *U* test was used for statistical analyses compared to the control group; ^*^p < 0.05.

For further characterization of the T cell response we determined the CD4/CD8 composition of the infiltrating T cells as well as the expression of the immune checkpoint molecule programmed cell death protein 1 (PD-1). In absolute numbers, T cells were pre-dominantly CD8+ T cells (Figure 4B). However, RTx combined with HHP vaccine particularly promoted CD4+ T cell infiltration (Figure 4A). Most tumor-infiltrating CD4+ T cells expressed PD-1 in response to RTx and combination of RTx with HHP vaccine enhanced it further significantly (Figure 4C). In contrast, the majority of tumor-infiltrating CD8+ T cells expressed PD-1 irrespective of the treatments (Figure 4C). When focusing on T cells of the peripheral blood of the mice, expression of PD-1 was observed only in very few CD4+ and CD8+ T cells (Figure 4D).

### Combination of RTx With Whole Tumor Cell-Based Vaccine Generated by HHP Induces Retardation of CT26 Tumor Growth in Balb/c Mice and Increases Their Survival

**Figure 5:**
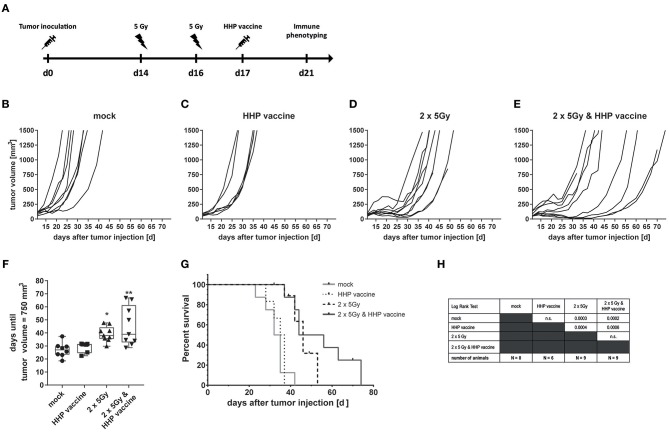
Impact of radiotherapy and whole tumor cell-based vaccine generated by HHP on CT26 colon carcinoma growth and survival. **(A)** 1.2 × 10^6^ viable CT26 cells were injected subcutaneously into syngeneic Balb/c mice. The tumor-bearing mice were either left untreated (mock), vaccinated at day 17 with 5 × 10^6^ CT26 tumor cells that had been inactivated with 200 MPa (HHP vaccine), locally irradiated at day 14 and 16 after tumor implantation with a single dose of 5Gy per fraction (2 × 5Gy), or treated with both RTx (2 × 5Gy) and HHP vaccine. The tumor growth is displayed as individual growth curves **(B–E)** and days until the tumor reached a volume of 750 mm^3^ **(F)**. The survival time of Balb/c mice after CT26 tumor implantation is depicted in **(G)**. When the tumor volume exceeded 1,500 mm^3^, the mice were euthanized. Significant values are determined by a Kruskal-Wallis test with Dunn's correction for multiple testing. The number of mice in the treatment groups, as well as the results of the Log-Rank (Mantel-Cox) test concerning the survival **(G)** is displayed in **(H)**; ^*^p < 0.05, ^**^p < 0.01.

To investigate if the efficiency of RTx in combination with HHP vaccination is only a melanoma-specific phenomenon, CT26 colon carcinoma-bearing Balb/c mice were treated similarly as the B16-F10 melanoma-bearing C57BL/6 mice (Figure 5A). RTx significantly retarded tumor growth compared to mock-treated or vaccinated mice. This was also reflected by the prolonged time until tumors reached a volume of 750 mm^3^ (Figures 5B–D,F). The combined treatment with RTx plus vaccination further delayed tumor growth in about half of the mice (Figure 5E). In accordance with that, the survival of the mice was further prolonged (Figures 5G,H).

Compared to B16-F10 tumors, the amount of tumor-infiltrating CD8+ T cells varied more (Supplemental Figure 2A), but a trend of reduced tumor size with higher infiltration of CD8+ T cells was observed (Supplemental Figure 2B). As seen in the B16-F10 model, most tumor-infiltrating CD4+ T cells expressed PD-1 in response to RTx, but combination with HHP vaccine did not enhance it further (Supplemental Figure 2C). Again, the majority of tumor-infiltrating CD8+ T cells expressed PD-1 irrespective of the treatment. But in contrast to B16-F10 tumors, RTx in combination with vaccination further slightly enhanced PD-1+ CD8+ T cells (Supplemental Figure 2C). As observed in the B16-F10 model (Figure 4D), only very view CD4+ and CD8+ T cells of the peripheral blood did express PD-1 (Supplemental Figure 2D).

## Discussion

Promising results have recently been achieved using immunotherapy such as checkpoint inhibitors to treat a range of different tumor entities. However, therapeutic cancer vaccines as sole immune therapy for solid cancer encounter three key challenges: immunogenicity of the vaccine, established diseases burden, and existing immune suppressive tumor microenvironment (30). Autologous whole tumor cell-based vaccines have the advantage that target antigens do not have to be prospectively identified and they deliver many TAAs, which are however aberrantly expressed self-antigens. In contrast to neo-antigens, the latter should only be able to activate remaining low affinity T cells and have to break self-tolerance. Several additional treatments have been developed and are discussed to overcome this hurdle, as e.g., repeated vaccination, addition of adjuvants or co-stimulators (31). Regarding the latter, RT might come into play. It has been demonstrated that besides immune suppressive mechanisms, ionizing radiation has additionally immune stimulatory priorities that enhance activation of DCs and improve antigen presentation, both being pre-requisites for induction of anti-tumor immune responses (16, 20, 32).

It was already demonstrated for many human cancer cells lines that HHP treatment with 200 MPa results in apoptotic and necrotic tumor cells that activate DCs following their phagocytosis (7, 21). Sipuleucel-T as therapeutic cancer vaccine against castration-resistant prostate cancer has been proven to show efficiency (33). This depicts that therapeutic vaccination with enriched DCs that are stimulated and loaded with antigen can work. In a pre-clinical setting, just recently Hradilova et al. demonstrated that HHP-killed lung cancer cell lines as source of TAAs in combination with the adjuvants poly(I:C) act as DC maturation signal. They further showed that DC-based HHP lung cancer vaccine generated from monocytes of NSCLC patients induces tumor-antigen specific CD8+ and CD4+ T cells (34). Currently a Phase I/II clinical trial for NSCLC is ongoing that uses DC-based active cellular immunotherapy (DCVAC/LuCa) in combination with chemotherapy and immune enhancers (NCT02470468). In an orthotopic mouse model of prostate cancer, the same group demonstrated that DC-based vaccines are as effective as chemotherapy to retard tumor growth. In this setting, however, no difference between un-pulsed DCs and those pulsed with HHP-killed tumor cells was seen. However, a tendency of increased numbers of CD8+ T cells and NK1.1 cells in the spleen of the animals was detected when DCs were pulsed with HHP-killed tumor cells (35).

We have aimed to focus on another cellular vaccine approach (36) using HHP-killed tumor cells alone as vaccine instead of tumor cell-loaded DCs. This approach aims to stimulate and deliver TAAs to DCs *in vivo* when the vaccine is combined with local stimulation of the tumor tissue by irradiation. A major mechanism for the observed synergistic effects is most likely that HHP-killed tumor cells are phagocytosed by the endogenous DCs and antigens are presented to T cells for T cell stimulation.

We here show that murine tumor cells, which are necessary to be applied if consecutive *in vivo* testing of multimodal therapies is performed in syngeneic mice (29), are killed in the same way as human tumor cells (7) by HHP. HHP-treatment was already included in the list of immunogenic cell death inducers (37). We additionally observed that murine tumor cells continue to degrade DNA following HHP-treatment. This might additionally impact on the immunogenicity of the vaccine, as it was already shown for DNA exonuclease Trex1 that it regulates RTx-induced immunogenicity of tumor cells (38, 39). Therefore, cytosolic DNA following treatment of the tumor cells with HHP might contribute via STING-dependent cytosolic DNA sensing to the immunogenicity of the HHP-vaccine (40). This has however to be proven in future work.

We focused on the new fact if RTx can be combined with syngeneic whole tumor cell-based vaccine without previous co-cultivation of the killed tumor cells with DCs and without any additional adjuvants. Combining vaccination with therapies that modify the tumor and its micro-environment should be promising approaches to enhance the vaccine's efficacy (41). We observed significantly reduced tumor growth and significantly improved survival of B16-F10 tumor-bearing C57BL/6 mice that had been treated with RTx plus HHP vaccine in comparison to RTx alone. Just vaccination did not impact on tumor growth and survival at all. In recent small phase I trials that combine vaccines with other immunotherapy evidence increases that boosting the immune system before vaccination can generate a better response (30). Targeting CTLA-4 in combination with a poxviral-based vaccine targeting prostate-specific antigen resulted in a small number of patients with increased frequency of antigen specific T-cells (42). In another phase I trial for prostate cancer, a vaccine containing two irradiated prostate cancer cell lines that express GM-CSF (GVAX-PCa) again in combination with targeting the immune suppressive immune checkpoint molecule CTLA-4 by ipilimumab, induced an increased expression of CD40 by DCs. This again suggests an enhanced DC function in these cancer patients (43). To exclude that a synergistic effect of RTx and HHP vaccination is only melanoma-specific, we additionally used the CT26 colon carcinoma model. In accordance with the B16-F10 tumor model, also CT26 tumor growth was further retarded when RTx was combined with HHP vaccination. This was seen in about half (4/9) of the mice.

Since the specific T cell numbers needed for an efficient cancer vaccine are unknown to date and do vary between tumor type, antigens and T cell receptor affinity (31), we here focused on analyses of number and quality of tumor-infiltrating immune cells following vaccination, RTx and combination of vaccination and RTx. The immune phenotyping data of B16-F10 tumors demonstrated an enhanced tumor infiltration of a variety of immune cells of the innate as well as the adaptive immune system after combination of RTx and the HHP vaccine. Although different immune cell subtypes are suspected to have diverse impact on tumor progression, the infiltration of immune cells is generally associated with good prognosis for melanoma patients (44) and for most of the solid tumors (45).

We revealed that combination of RTx with HHP vaccine generates a favorable anti-tumor immune microenvironment for melanoma. γδT cells are known to infiltrate into melanoma and are capable of killing melanoma cells (46). We identified that only combination of RTx with HHP vaccine increased the number of γδT cells in the tumor. Further, NK cells were significantly enhanced. These innate immune cells are key players in mediating anti-tumor immunity (47). We also previously observed that NK cell depletion after immunization results in a significant acceleration of melanoma growth (48). NKT cells were also significantly enhanced and may lead to downstream activation of both innate and adaptive immune cells in the tumor microenvironment (49). Since B cells might foster tumor-promoting humoral immunity in melanoma (50), decreased numbers following RTx plus HHP vaccine treatment should also contribute to a beneficial therapy-induced tumor microenvironment.

They et al. demonstrated that a favorable modulation of the melanoma microenvironment fosters the infiltration of CD4+ and CD8+ T cells (51). However, tumor escape by upregulation of PD-1 is frequent and additional treatment with anti-PD-1 antibody restored effector functions of CD4+ and CD8+ T cells as well as of NK cells and γδT cells. We demonstrate that combination of RTx with HHP vaccine also fosters infiltration of CD4+ and CD8+ T cells as well as that of NK cells and γδT cells into B16-F10 melanomas. In the CT26 model, combination of RTx plus HHP vaccine resulted in heterogeneously responding tumors. Here, high CD8+ T cell infiltration tended to result in smaller tumors (Supplemental Figure 2). We further observed a high expression of PD-1 on infiltrating T cells. This depicts both that activation of the T cells against the tumor has taken place and that subsequently immune suppressive checkpoint molecules such as PD-1 are expressed to regulate the immune response and that re-stimulation of the immune system by anti-PD-1 treatment will be necessary. Dyck et al. demonstrated in the CT26 model that anti-PD-1 treatment reduced regulatory T cell induction and enhanced CD8+ T cell mediated tumor killing. Combined treatment of tumor-bearing mice with a vaccine, comprising heat-shocked irradiated tumor cells and a TLR 7/8 agonist, significantly reduced tumor growth and enhanced survival (52). This calls for further improvement of induction of anti-tumor immune responses by combining RTx plus HHP vaccine with immune checkpoint-inhibition in the future (16, 18, 53). While almost absent in blood, the majority of CD8+ T cells infiltrating into B16 tumors and almost all CD8+ T cells in CT26 tumors expressed PD-1. This enrichment of PD-1+ T cells in the tumor was already reported for patients with metastatic disease (54), indicating that the up-regulation of the inhibitory receptor PD-1 is driven by the tumor microenvironment. Nevertheless, PD-1 expression can also be considered as favorable marker for an effectively primed T cell response, as suggested by Fernandez-Poma et al. Only the fraction of T cells selected for positive PD-1 expression exhibited anti-tumor reactivity when adoptively transferred into mice and combination with anti-PD-L1 further enhanced tumor control (55).

Future work will focus on a triple combination of RTx with HHP vaccination and checkpoint inhibition for the induction of anti-tumor immune responses to primary and abscopal tumors (16). Furthermore, one should think about to modify the radiation dose that has most likely to be adapted very individually in the future for optimization of immune stimulation by RTx. However, one has to be aware that too high single dose might again decrease immunogenicity of the tumors (38). Additionally, the HHP-vaccine could be injected multiple times to break self-tolerance with appropriate adjuvants. We demonstrated in another pre-clinical setting that repeated vaccination of tumor cells that had been killed by RTx in combination with heat is superior to single vaccination with regard to induction of tumor growth retardation (48). Even though many hurdles still will have to be overcome for most beneficial combination of RTx with tumor cell-based vaccines, such approaches are particularly important for patients who harbor weak spontaneous immune responses to their cancer. Furthermore, development of cancers vaccines have to respect that standard of care for most cancer patients involves chemotherapy and/or RTx (31). The here presented pre-clinical work give first hints that RTx is well-combinable with tumor-cell based vaccines generated by HHP and provides a basis for continuing work on optimization of multimodal cancer therapies.

## Ethics Statement

All animal experiments were conducted according to the guidelines of the Federation of European Laboratory Animal Science Associations (FELASA) and the Gesellschaft für Versuchstierkunde (GV-SOLAS) and were authorized by the government of Mittelfranken/Unterfranken.

## Author Contributions

CS carried out most of the *in vitro* experiments and parts of the *in vivo* work and wrote the manuscript together with MR, UG, and BF. MR carried out most of the *in vivo* work and wrote the manuscript together with CS, BF, and UG. LD contributed to the *in vivo* experiments and to the drafting of the manuscript. E-MW contributed to drafting the *in vitro* experiments and parts of the Balb/c *in vivo* experiments. SU performed parts of the *in vitro* experiments. MI optimized the HHP treatment procedure together with NE. ES drafted the vaccine preparation experiments together with BF. RF contributed to the design of the work. UG drafted the whole study including most of the *in vitro* and *in vivo* experiments together with BF. UG further drafted the manuscript and wrote it together with BF, CS, and MR. BF drafted the whole study including most of the *in vitro* and *in vivo* experiments together with UG. BF further wrote the manuscript together with UG, CS, and MR. All authors read and approved the manuscript.

### Conflict of Interest Statement

The authors declare that the research was conducted in the absence of any commercial or financial relationships that could be construed as a potential conflict of interest.

## Supplementary Material

Supplemental Figure 1 Technical sketch of the high hydrostatic pressure aperture that was used for the generation of the tumor cell-based vaccine. The numbered properties of the aperture are explained in the main text.

Supplemental Figure 2 Impact of radiotherapy and HHP vaccination on infiltration of T cells into CT26 tumors and on PD-1 expression of T cells. Single cell suspensions of tumors from mice that had been treated with RTx (2 × 5Gy) and/or HHP vaccine (CT26 tumor cells that had been inactivated with 200 MPa) were prepared on day 21 after tumor inoculation. Multicolor flow cytometry was performed to detect tumor infiltrating CD4+ and CD8+ T cells **(A)**. The infiltration of CD8+ T cells into the tumor in relation to the tumor weight is depicted in **(B)**. Further, expression of PD-1 on CD4+ and CD8+ T cells being present in the tumors **(C)** and those circulating in blood **(D)** is shown. Data are presented as box plots showing the median and minimum to maximum values. *n* = 6; Mann-Whitney *U* test was used for statistical analyses; ^*^*p* < 0.05.

## Abbreviations

AnxA5: AnnexinA5
CTLA-4: cytotoxic T-lymphocyte-associated Protein 4
DC: dendritic cell
FELASA: Federation of European Laboratory Animal Science Associations
GM-CSF: granulocyte-macrophage colony-stimulating factor
GV-SOLAS: Gesellschaft für Versuchstierkunde
HHP: high hydrostatic pressure
HLA: human leukocyte antigen
NK: natural killer
PI: propidium iodide
PD-1: programmed cell death protein 1
RTx: radiotherapy
SD: standard deviation
SEM: standard error of the mean
TAA: tumor-associated antigen
TIL: tumor-infiltrating leukocytes.
