# Supplementary figures and images for: Tumor Cell-Based Vaccine Generated With High Hydrostatic Pressure Synergizes With Radiotherapy by Generating a Favorable Anti-tumor Immune Microenvironment
Source: Front Oncol. 2019 Aug 28;9:805. doi: 10.3389/fonc.2019.00805 (PMC6722191; doi:10.3389/fonc.2019.00805)

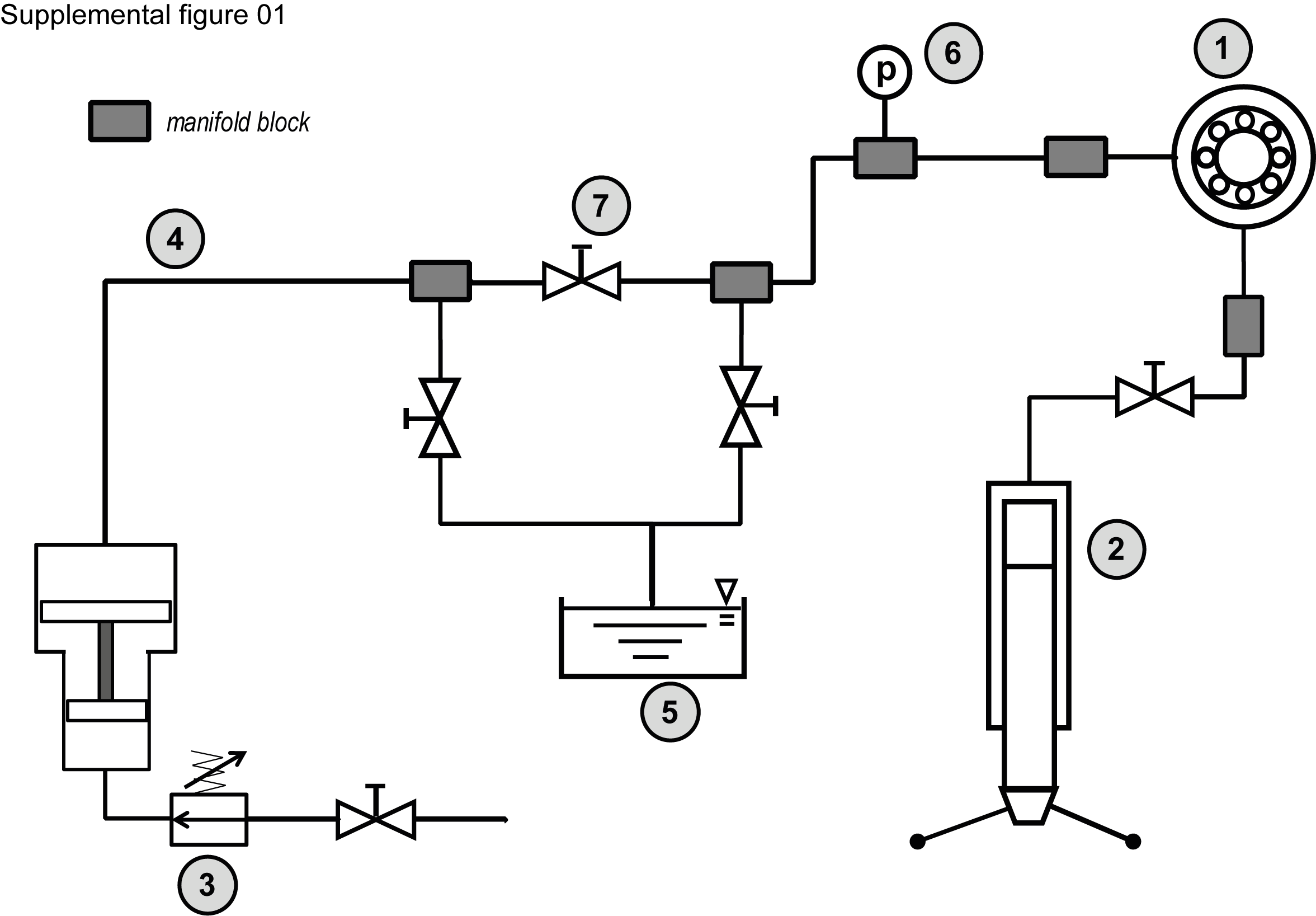

Supplement: Supplemental Figure 1 — Technical sketch of the high hydrostatic pressure aperture that was used for the generation of the tumor cell-based vaccine. The numbered properties of the aperture are explained in the main text. [file Image_1.TIF]

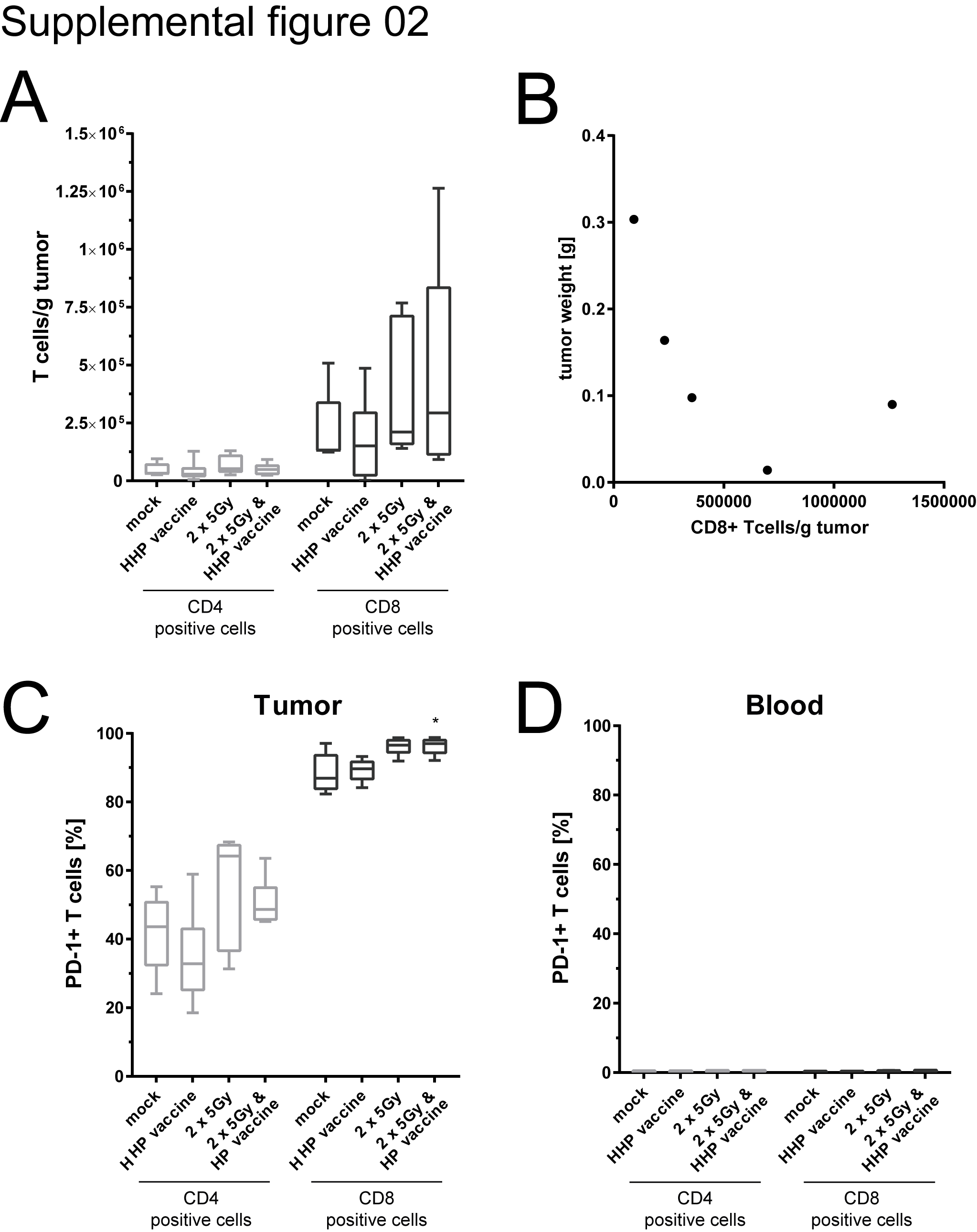

Supplement: Supplemental Figure 2 — Impact of radiotherapy and HHP vaccination on infiltration of T cells into CT26 tumors and on PD-1 expression of T cells. Single cell suspensions of tumors from mice that had been treated with RTx (2 × 5Gy) and/or HHP vaccine (CT26 tumor cells that had been inactivated with 200 MPa) were prepared on day 21 after tumor inoculation. Multicolor flow cytometry was performed to detect tumor infiltrating CD4+ and CD8+ T cells (A). The infiltration of CD8+ T cells into the tumor in relation to the tumor weight is depicted in (B). Further, expression of PD-1 on CD4+ and CD8+ T cells being present in the tumors (C) and those circulating in blood (D) is shown. Data are presented as box plots showing the median and minimum to maximum values. n = 6; Mann-Whitney U test was used for statistical analyses; *p < 0.05. [file Image_2.TIF]
